# Sociodemographic characteristics of patients with children in a methadone maintenance program: a cross-sectional study

**DOI:** 10.1186/s12954-019-0283-9

**Published:** 2019-02-11

**Authors:** Candice Luo, Nitika Sanger, Laura Zielinski, Meha Bhatt, Hamnah Shahid, Ieta Shams, Natalia Mouravska, Sabrina Luetam, Jackie Hudson, Lehana Thabane, Zainab Samaan

**Affiliations:** 1 Bachelor of Health Sciences Program, McMaster University, 1280 Main St. W., Hamilton, Ontario L8S 4L8 Canada; 2 Medical Sciences Graduate Program, McMaster University, 1280 Main St. W., Hamilton, Ontario L8S 4L8 Canada; 3 MiNDS Graduate Program, McMaster University, 1280 Main St. W., Hamilton, Ontario L8S 4L8 Canada; 4 Department of Health Research Methods, Evidence, and Impact, McMaster University, 1280 Main St. W., Hamilton, Ontario L8S 4L8 Canada; 5 Arts and Science Program, McMaster University, 1280 Main St. W., Hamilton, Ontario L8S 4L8 Canada; 6 Department of Psychiatry and Behavioural Neurosciences, McMaster University, 1280 Main St. W., Hamilton, Ontario L8S 4L8 Canada; 7 Hamilton Health Sciences, 237 Barton St. E., Hamilton, Ontario L8L 2X2 Canada; 8 Michael G. DeGroote School of Medicine, McMaster University, 1280 Main St. W., Hamilton, Ontario L8S 4L8 Canada; 9 Peter Boris Centre for Addictions Research, St Joseph’s Healthcare Hamilton, 100th West 5th St., Hamilton, Ontario L8N 3K7 Canada; 10 Populaton Genomics Program, Chanchlani Research Centre, McMaster University, 1280 Main St. W., Hamilton, Ontario L8S 4L8 Canada; 11 Mood Disorders Program, St. Joseph’s Healthcare, 100 West 5th St., Hamilton, Ontario L8N 3K7 Canada

**Keywords:** Cross-sectional, Methadone, MMT, Opioid, Behaviors, Parental

## Abstract

**Background:**

Ever-increasing numbers of opioid use disorder (OUD) in Canada has created the recent opioid crisis. One common treatment for OUD is methadone maintenance treatment (MMT). Various factors, including being a parent which entails specific stressors, may increase susceptibility to negative treatment outcomes. This study aims to investigate differences between OUD patients with and without children in socio-demographic and clinical outcomes.

**Methods:**

Data for this study are part of a larger program. All participants are 18+ years old with OUD, provided consent, and receiving MMT. We performed a multivariable logistic regression to examine the differences between participants’ parental status, sociodemographic variables, and clinical parameters including MMT outcomes. We performed subgroup analyses on individuals with children younger than 18.

**Results:**

A total of 1099 participants were included, with 64% having children. Participants with children were older (OR 1.06, 95% CI 1.04, 1.08), more likely to be female (OR 2.39, 95% CI 1.75, 3.27), living with a partner (OR 1.75, 95% CI 1.27, 2.41), first exposed to opioids through a prescription (OR 1.517, 95% CI 1.13, 2.04) and had lower levels of education (OR 1.86, 95% CI 1.20, 2.87). There was no significant difference in illicit opioid use patterns between groups. Same results held true in the subgroup analyses based on the age of the children except for participant age.

**Conclusion:**

Our results demonstrate social and demographic differences between parents and non-parents receiving MMT. These differences highlight the need to understand necessary additional support for parents such as child support and other necessary therapies.

## Introduction

Prescription opioids (PO) are medications used for treating illnesses including chronic pain and opioid use disorder (OUD) [1]. From 2000 to 2004, the prevalence of Canadian opioid users increased steadily, and in 2013, about 5.9% of the population reported misusing PO [2, 3]. To combat OUD, methadone maintenance treatment (MMT) is commonly used as an opioid substitution therapy due to its availability, government subsidization, and higher efficacy compared to abstinence or solely psychosocial treatment [4, 5]. In Ontario alone, over 50,000 adults were enrolled in MMT in 2013, making MMT the most commonly used opioid substitution therapy [3]. Binding of methadone to receptors significantly decreases withdrawal symptoms and cravings for other opioids by acting as a long-term agonist to these receptors and as an antagonist to the *N*-methyl-d-aspartate neural receptors [1, 6]. The effects of methadone prevent the user from experiencing the euphoria associated with stronger illicit opioids, decreasing further use [1, 6].

Despite MMT’s effectiveness, studies have shown a 30 to 70% rate of relapse in MMT patients with little research investigating factors explaining such high rates [7, 8]. We speculate this may be because patients on MMT often have a higher number of psychosocial comorbidities that can impact their success in MMT [9]. In addition, many randomized controlled trials that tested the effectiveness of opioid substitution therapies including methadone excluded patients with comorbid disorders leading to various rates of response compared to other types of study designs such as observational studies [10]. Despite studies investigating the efficacy of MMT in the general population [11, 12], there has also been a limited number of studies exploring the influence of parental responsibilities on MMT outcomes.

Undoubtedly, parents are one group of patients with unique social circumstances that may influence their treatment needs or outcomes. Factors include being a parent which comes with stressors and obligations unique to their life that may increase susceptibility to various negative health behaviors. Such stressors may include providing children with the necessities of life, paying larger bills, physical obligations of child care, and higher living expenses. These may result in parents having to work longer hours, being more distressed, and relieving stress with drugs. Although previous studies have examined the role of parental responsibilities on illicit drug use broadly [13, 14] as well as the quality of parenting in MMT patients [15, 16], to our knowledge, no study has explored social demographic variables specifically within the parenting population.

Parents have the responsibility of ensuring the well-being of the child, which comes with numerous daily tasks and considerations. This makes them a unique population within MMT population, thus requiring special considerations of their social demographic situation. Parental responsibilities, such as supervising their child’s behavior, have been reported to be a source of stress for parents [17]. Although physiological and psychological stress have been reported to influence MMT outcomes negatively [18], parents also have been shown to have intrinsic motivation to ensure their child’s safety and wellbeing [19]. Concern over their child’s wellbeing has been shown to be a motivating factor for parents to enter treatment [20]. However, one factor that may discourage parents from entering treatment is avoiding separation from their child due to suspected lack of competency in their child caring duties [21]. Therefore, parents without stable incomes and family support might be more inclined to use other substances or relapse back into illicit opioid use to relieve their overwhelming stress from these responsibilities.

Research has shown conflicting evidence on parental quality as well as addiction treatment outcomes in this population [13, 22, 23]. Studies have found that bearing child care responsibilities negatively affected various treatment outcomes [13]. Some outcomes discussed in the literature include addiction severities as well as psychiatric symptoms [9, 22]. However, studies have found that parents with the community and social support were able to increase MMT adherence and long-term benefits, while lowering the rate of polysubstance use and addiction severities [14, 23–26]. It is important to highlight, however, that these studies lacked specificity to an opioid-using or MMT population. Therefore, it is crucial to explore the current social demographic circumstances of the MMT population to gain a comprehensive understanding of their circumstance.

Current literature supports the notion that additional support from various agencies may further motivate parents to adhere to treatment [14, 23–26]. This assistance may be in the form of familial support, social services, and additional treatment for their comorbidities, among others [14, 23–26]. Further investigating the differences between the two populations may elucidate specific needs within this population not known prior. This may benefit clinicians to better evaluate treatment plans, as well as provide evidence for program designers to ensure MMT programs meet the needs of this patient population. The present cross-sectional study aims to compare social and demographic differences between parents and non-parents with opioid use disorder to further understand patient needs in order to better tailor treatment for them.

The present study’s objectives are as follows:

1. Determine the social and demographic differences between participants with children compared to those without children currently on MMT.
2. Determine the social and demographic differences between participants with children under the age of 18 compared to those without children currently on MMT.

## Methods

**Fig. 1:**
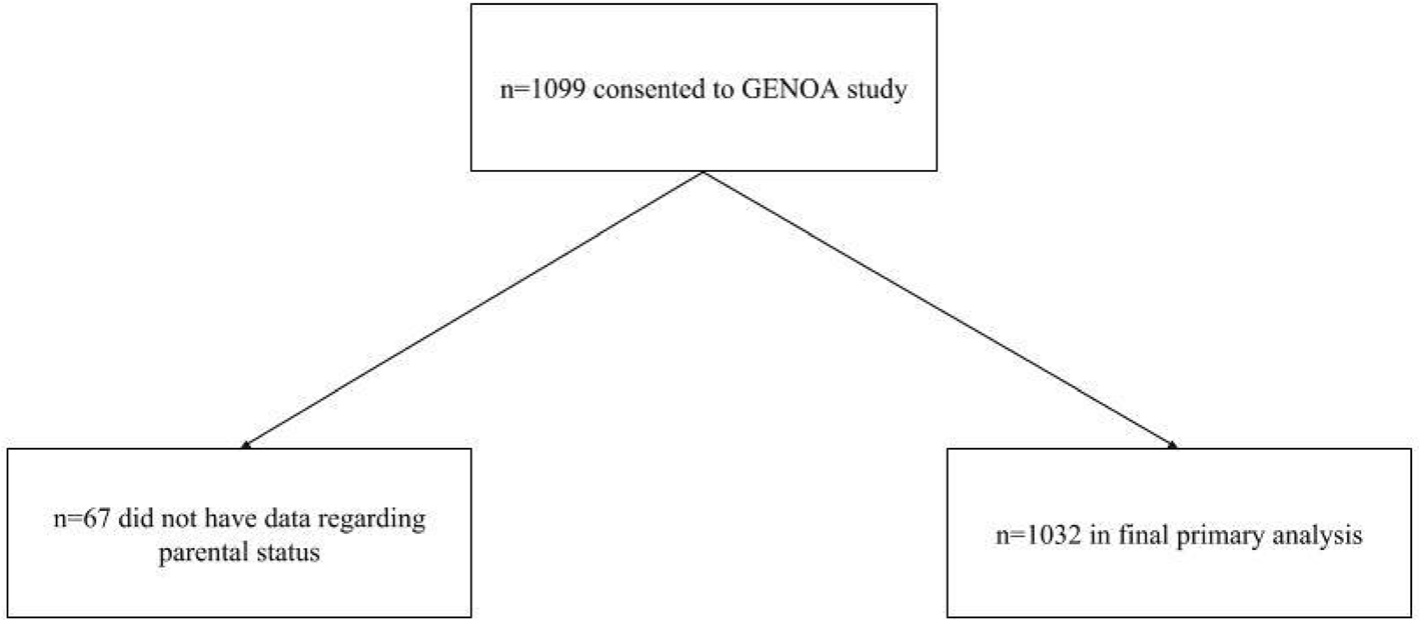
Participant flow diagram

In this study, data were retrieved from an ongoing prospective study, the Genetics of Opioid Research (GENOA) research program, conducted in collaboration with the Population Genomics Program at McMaster University and Canadian Addiction Treatment Centres (CATC) across Ontario. Participants were required to be 18 years of age or older, currently receiving MMT for OUD, provided information regarding parental status, and provided a written informed consent. A detailed description of the GENOA protocol can be found elsewhere [27]. The exclusion criteria for participants included the inability to communicate in English (Fig. 1). We excluded participants from this study with missing data regarding parental status. None of the study participants had missing urine toxicology screens.

Data were collected via baseline questionnaires administered to patients from June 2013 to October 2016. Case report forms were used to collect demographic information including parental status, age, sex, duration of opioid use, onset of methadone treatment, age of youngest child, and other substance use in the last month. Methadone dose was self-reported during the baseline interview and was further verified by checking the medical records as per the study procedures and participants’ consent to check the medical records. Although there was no exclusion criterion for the duration on methadone, for example, to reach a stable dose, the average duration in months was 47 months for participants on MMT included in our analysis. Substance use was self-reported using the Maudsley Addiction Profile instrument which has been described previously [28]. The current study extracted data from 1099 participants who completed the baseline screening questionnaires. Parental status was collected from the baseline questionnaire. Specifically, we asked participants the number of children they had, and the ages of the youngest and oldest children.

Participants were followed up for 3 months with weekly or bi-weekly urine drug screens for illicit opioid use. We define treatment outcomes as illicit opioid use measured by any positive opioid urine screens during the 3-month period of the study. Patients with at least one positive opioid urine screen (excluding methadone) were coded positive in illicit opioid use, while patients with no positive opioid urine screens were coded as negative.

### Statistical analysis

All statistical analyses were completed using SPSS 23.0 software. For baseline characteristics, means and standard deviations were reported for continuous variables and percentages were reported for dichotomous variables. Continuous variables include age, amount of the last dose, and length of MMT treatment. Dichotomous variables include sex and parental status. Participants with missing values for either their parental status or positive opioid urine screens were excluded from this analysis, resulting in a final model with 1032 participants. Using the rule of 10 events per predictor, with a power of 80%, and alpha of 5%, our study is sufficiently powered to answer our primary question with 12 predictor variables in the main model [29].

A multivariable logistic regression analysis was used to determine the association between having children and varying social factors. We coded parental status, illicit opioid use, employment status, smoking status, and marital status dichotomously. Parental status was coded as either having children or not having children. Illicit opioid use was coded as having used any illicit opioids or having not used any illicit opioids at baseline. Employment status was coded as being currently employed in the last 30 days, or not employed in the last 30 days. Smoking status was coded as current smoker versus former smoker/having never smoked. Marital status was coded as being married, in common law relationships or living with a partner, or divorced, separated, or never married.

We performed a sub-group analysis in participants with children under 18 years of age to test whether parents of younger children have differing social situations. We coded all variables identical to the main regression. We then ran a multivariable logistic regression examining the same factors as the first regression.

Using the variance inflation factor (VIF), we assessed for collinearity and variables with VIF > 10 were subsequently excluded from all analyses. We reported adjusted odds ratio (aOR), 95% confidence intervals (CI), and *p* values. A two-sided *p* value of 0.05 or less was considered significant.

Strengthening the Reporting of Observational Studies in Epidemiology (STROBE) guidelines were used to ensure standardized reporting in this study [30].

## Results

### Baseline characteristics

Baseline characteristics are displayed in Table 1. Approximately 64.3% of participants indicated that they had children, with approximately 67.8% of the parenting population with children below the age of 18. The mean age of the participants with children was 41.2 (SD 10.6) years, only 2.1 years older than the average age of participants without children (SD 10.5). Participants with children tended to have less years of education (47.2% of participants with children completed high school vs. 54.8% of participants without children completed high school), more likely to be unemployed (69.7 vs. 59.6%), were currently with a partner (36.1 vs. 24.3%), and first introduced to opioids through a physician prescription (54.6 vs 35.7%). Participants with children also had a longer MMT treatment duration (mean: 51.9 months, SD: 51.6) compared to participants without children (mean: 39.4, SD: 41.6).

**Table 1:** Patient demographics

	Have children (*n* = 707)	No children (*n* = 392)	*P* value
Sex, *n* (%)			
Male	341 (48.2)	259 (66.1)	< 0.0001
Age (years), mean (SD)	41.2 (10.6)	39.1 (10.5)	0.109
Highest level of education, *n* (%)			
Grade 8 and below	175 (24.8)	64 (16.5)	0.001
Grades 9–12	332 (47.2)	213 (54.8)	0.019
College/university, trade school, masters/PhD	196 (27.7)	112 (28.6)	0.779
Employment status, *n* (%)			
Currently employed	214 (30.3)	158 (40.4)	0.001
Marital status, *n* (%)			
Married/common law/living with partner	255 (36.1)	95 (24.3)	< 0.0001
Source of first opioid exposure, *n* (%)			
Prescribed by physician	386 (54.6)	140 (35.7)	< 0.0001
Smoking status, *n* (%)			
Current smoker	597 (84.6)	335 (85.4)	0.344
Self-reported substance use in the past 30 days, *n* (%)*			
Alcohol	272 (38.4)	179 (45.7)	0.024
Benzodiazepine	71 (10.0)	51 (7.2)	0.134
Heroin	78 (11.0)	61 (15.6)	0.046
Cocaine	117 (16.5)	84 (21.4)	0.051
Amphetamine	45 (6.4)	27 (6.9)	0.799
Crack cocaine	81 (11.5)	42 (10.7)	0.765
Cannabis	344 (48.6)	235 (59.9)	0.000
Illicit methadone	12 (1.7)	3 (0.8)	0.280
Other drugs**	361 (51.1)	216 (55.1)	0.208
Methadone dose (mg/day), mean (SD)	76.0 (46.3)	71.0 (42.9)	0.223
Length of current treatment (months), mean (SD)	51.9 (51.6)	39.4 (41.6)	< 0.0001
Age of first opioid use, mean (SD)	26.6 (9.1)	22.8 (7.6)	< 0.0001
Illicit opioid use through urine screens, *n* (%)			
At least 1 positive urine screen during 3 month study period	395 (55.9)	214 (54.6)	0.704

*Some people use more than 1 substance at once so the values of self-reported substance use might not add up to 100% in each row

**Other drugs used by participants include ketamine, Percocet, diluadid, oxycontin, fentanyl, oxycodeine, hydromorphone, morphine, acetaminophen, crystal meth, gabapentin, MDMA, OxyNeo, Ritalin, poppy seed opium, hashish, illicit Suboxone, and codeine

### Primary logistic regression

As seen in Table 2, we ran a multivariable logistic regression comparing participants with children and participants without children. Parents were shown to be more likely to be older (aOR for each additional year in age: 1.058, 95% CI 1.039, 1.078, *p* < 0.0001), more likely to be female (aOR 2.394, 95% CI 1.753, 3.268, *p* < 0.0001), had an education level of grade 8 (equivalent to 8–10 years of schooling) or below (aOR 1.856, 95% CI 1.199, 2.872, *p* = 0.005), more likely to be married or in a common law relationship, or living with a partner (aOR 1.750, 95% CI 1.273, 2.406, *p* < 0.0001), and more likely to have first been exposed to opioids through a physician prescription (aOR 1.517, 95% CI 1.129, 2.038, *p* = 0.006).

**Table 2:** Primary logistic regression comparing sociodemographic outcomes between participants without children and participants with children on MMT

	aOR	95% CI		*P* value
		Lower bounds	Upper bounds	
Sex (male)	2.417	1.775	3.292	< 0.0001
Age (years)	1.060	1.040	1.079	< 0.0001
Highest level of education				
Grade 8 and below	1.865	1.209	2.877	0.005
Grades 9–12	1.171	0.837	1.638	0.358
College/university, trade school, or masters/PhD	0.878	0.618	1.246	0.465
Employment status	0.817	0.597	1.118	0.207
Marital status				
Married/common law/living with partner	1.766	1.288	2.422	< 0.0001
Source of first opioid exposure				
Physician prescribed	1.517	1.129	2.038	0.006
Smoking status				
Current smoker	0.962	0.637	1.452	0.853
Self-reported substance use in the past 30 days				
Alcohol	0.935	0.695	1.259	0.658
Benzodiazepine	0.771	0.470	1.263	0.301
Heroin	0.935	0.695	1.527	0.789
Cocaine	0.858	0.574	1.281	0.453
Amphetamine	0.935	0.458	1.910	0.854
Crack cocaine	1.370	0.791	2.373	0.262
Cannabis	0.872	0.650	1.170	0.361
Illicit methadone	3.662	0.753	17.819	0.108
Other drug*	0.982	0.733	1.315	0.902
Methadone dose (mg/day)	1.000	0.999	1.003	0.941
Length of current treatment (months)	1.000	0.997	1.004	0.999
Age of first opioid use	1.020	0.999	1.043	0.068
Illicit opioid use by urine screen At least 1 positive urine screen during 3-month study period	1.111	0.814	1.514	0.508

*aOR* adjusted odds ratio, *CI* confidence intervals, *parental status* having one or more children, reference group is being a parent, substance use is not an indication of substance use disorder

*Other drugs used by participants include ketamine, Percocet, diluadid, oxycontin, fentanyl, oxycodeine, hydromorphone, morphine, acetaminophen, crystal meth, gabapentin, MDMA, OxyNeo, Ritalin, poppy seed opium, hashish, illicit Suboxone, and codeine

### Subgroup 1: Parents with children at or below 18 years of age

From our total sample of 1032, 446 participants had children younger or at the age of 18. We ran a multivariable logistic regression adjusting for duration of MMT and methadone dose. Results showed that parents with children under the age of 18 were much more likely to be female (aOR 2.201, 95% CI 1.601, 3.025, *p* < 0.0001), married or in a common law relationship or living with a partner (aOR 1.826, 95% CI 1.317, 2.533, *p* < 0.0001), have an education level below grade 8 which is equivalent to 8–10 years of schooling (aOR 1.805, 95% CI 1.137, 2.866, *p* = 0.013), more likely to have first been exposed to opioids through a physician prescription (aOR 1.540, 95% CI 1.132, 2.094, *p* = 0.006) and likely to be older when they first started opioids (aOR 1.028, 95% CI 1.003, 1.053, *p* = 0.029) (Table 3).

**Table 3:** Subgroup of parents with children under 18 compared to the population without children

	aOR	95% CI		*P* value
		Lower bounds	Upper bounds	
Sex (male)	2.231	1.627	3.060	< 0.0001
Age (years)	1.012	0.990	1.033	0.283
Highest level of education				
Grade 8 and below	1.787	1.129	2.829	0.013
Grades 9–12	1.139	0.803	1.617	0.465
College/university, trade school, or masters/PhD	0.855	0.612	1.199	0.365
Employment status	0.817	0.578	1.110	0.183
Marital status				
Married/common law/living with partner	1.826	1.317	2.533	< 0.0001
Source of first opioid exposure				
Physician prescribed	1.540	1.132	2.094	0.006
Smoking status				
Current smoker	1.050	0.675	1.634	0.827
Self-reported substance use in the past 30 days				
Alcohol	0.950	0.698	1.292	0.742
Benzodiazepine	0.680	0.398	1.160	0.157
Heroin	0.917	0.550	1.527	0.738
Cocaine	0.846	0.559	1.281	0.430
Amphetamine	1.107	0.540	2.269	0.781
Crack cocaine	1.256	0.697	2.263	0.449
Cannabis	0.826	0.608	1.123	0.222
Illicit methadone	4.464	0.915	21.769	0.064
Other drug*	0.970	0.715	1.316	0.847
Methadone dose (mg/day)	1.000	0.995	1.002	0.506
Length of current treatment (months)	1.000	0.996	1.004	0.924
Age of first opioid use	1.028	1.003	1.053	0.029
Illicit opioid use by urine screens At least 1 positive urine screen during 3 month study period	1.108	0.803	1.529	0.534

Included in analysis: 838

*aOR* adjusted odds ratio, *CI* confidence intervals, *parental status*: having one or more children, reference group is being a parent, substance use is not an indication of substance use disorder

*Other drugs used by participants include ketamine, Percocet, diluadid, oxycontin, fentanyl, oxycodeine, hydromorphone, morphine, acetaminophen, crystal meth, gabapentin, MDMA, OxyNeo, Ritalin, poppy seed opium, Hashish, illicit Suboxone, and codeine

## Discussion

The present study suggests differing social and demographic circumstances between the parenting population and the non-parenting population on MMT. Parental status does not influence MMT outcomes, contrary to results from previous studies [13, 31, 32]. In our study, we did not find a statistically significant difference in illicit opioid screens, length of treatment, nor average methadone dose between parents and non-parents. We found age, sex, education, marital status, and physician prescription as first exposure to opioids to be statistically significant in both the main regression and the subgroup analysis. We also found the age of first opioid exposure to be statistically significant in our subgroup analysis. There are a variety of factors that may result in this population being more likely to be first exposed through a physician prescription, such as persistent pain after labor and delivery [33]. These results suggest that individuals with children are demographically different and more likely to receive opioids through a prescription; therefore, they may have different risk factors, such as increased parenting responsibility that can increase daily stress levels or physical stress unique to parents that can impact their likelihood of opioid use disorder requiring special attention from health care providers. However, it must be recognized that this study cannot provide conclusive evidence that having children is a risk factor for opioid use disorder and can only highlight demographic differences in first exposure to opioids.

There has been a limited number of studies exploring the parenting population in MMT specifically. Although previous studies have examined the role of parental responsibilities on illicit drug outcomes [13, 24, 26] as well as exploring the quality of parenting in MMT patients [15] to our knowledge, this is the largest cohort study done investigating population differences between parents and non-parents within a *Canadian* context. As well, there are methodological differences between previous studies and ours that should be considered. A similar study investigated the influence of parental responsibilities in substance use treatment for residential programs and MMT outcomes and found a general negative association between parental status and various treatment outcomes [13]. However, they do not account for important confounding variables such as the length of the treatment duration, and the dose of methadone provided, both of which have both been found to be important factors in the success of treatment previously [16]. Other studies examined substance use among young parents such as alcohol abuse and patterns of their experiences with child welfare services [24, 26]. Although these studies highlight important considerations in parents with substance use disorder, they lack the comparison between the two patient populations (i.e., patients with children and patients without children) and other social factors that play a role in risk factors for substance use.

In our study, we have shown that parents differ in various sociodemographic categories that may affect their susceptibility to substance use disorder, quality of life, and future treatment outcomes or prognosis. However, there is uncertainty as to whether parents with differing child care responsibilities may present differing vulnerabilities to various risk factors compared to parents without any child care responsibilities. Patients with children on MMT have traditionally been shown to be less involved in parenting responsibilities [34, 35]. Specifically, Suchman and Luthar (2000) reported that mothers with substance use disorders with children under 16 years of age had lower parental involvement in child-caring activities compared to mothers without substance use disorders [34]. Mothers on MMT with children in child care services had lower methadone dose intakes compared to controls, which was not supported by our results [35–37]. Likewise, research has also shown that women with parenting responsibilities involved in treatment have more psychiatric symptoms than those without parenting responsibilities [34]. This highlights the need to further examine and evaluate treatment needs for those with children. With high rates of physician prescription opioids in Ontario [38], it is unsurprising that patients with children are significantly more likely to be first exposed to opioids through physician prescriptions than those without children. With the negative outcomes of OUD, and its’ impact on parenting quality, clinicians should be more aware of the potential dangers of prescribing opioids without careful management. As well, chances of obtaining OUD and its’ potential negative impact on parenting abilities should be highlighted on guidelines as an essential point of discussion with patients during informed consent.

In this study, we found that patients with children are more likely to be women compared to patients without children; however, this population may not represent all fathers with OUD because fathers on MMT may be overlooked in treatment. The literature has shown that fathers are more likely to be using opioids at the time of their child’s birth, less likely to be employed, and have relatively poorer vocational status [39, 40]. As well, fathers with OUD are found to be less likely to be the legal guardian of their child, to be living in the same household as the child, and providing financial support [39]. This may explain why we observed higher numbers of females with children in this study.

### Strengths and limitations

Our study has a number of strengths. Previous research fails to take into account important confounding factors such as the length of treatment as well as participant’s methadone dose into their analysis [39]. As well, they only conducted a univariate analysis and potentially further subjecting their results to a risk of confounding factors [39, 41]. Our study is unique in that we investigated social demographic differences between the parenting population, examining a wide array of factors that may be associated with a risk of opioid use disorder. In addition, our larger sample is more representative of the OUD population compared to smaller studies that may be at a higher risk of a type I error. With a large sample size, we are more confident in addressing our research question. Furthermore, our data were collected from a heterogeneous sample from various socioeconomic environments. This increases the generalizability of our findings to the general population in Southern Ontario. Lastly, we measure illicit opioid use through urine screens as opposed to self-reports, thus minimizing chances of misclassification bias in our study compared to previous studies that used self-reported illicit opioid use [42–44].

There are limitations of our study that should be mentioned. Our study is a cross-sectional design. Therefore, it is not possible to evaluate causation of parental status on any of the sociodemographic variables or treatment outcomes assessed. Second, we did not characterize the extent of the parental responsibilities in participants with children. We recognize that many parents with children in MMT may not be living with their children, and thus, do not carry any added parental responsibilities. Also, we did not measure the amount of support they were receiving, and whether it was spousal, social, or familial support. Furthermore, our recruitment process is through a volunteer basis. Therefore, our sample may be subjected to volunteer bias. Finally, our study may not be generalizable to populations in other provinces or countries, as we only extracted data from participants who attended MMT clinics in Southern Ontario.

Despite our limitations, our study highlights the social demographic differences between well-characterized parents on MMT. These differences may be used by services to improve their assessment and support of patients with children.

## Conclusion

The present study explores the social demographic differences between the parenting population and the non-parenting population in MMT, something that is noticeably absent in the literature. Our findings indicate that parents tend to be different than their counterpart in sex, education level, employment status, more likely to be physician prescribed, and started using opioids at an older age. We also found that parents with younger children have similar social and demographic situations compared to all parents. Future research should focus on the relationship between risk factors for OUD and the extent of child care responsibilities for the MMT population. As a society, it is imperative that we generate an environment that is conducive for the healthy development of children. We hope that our findings will support researchers in the further development of MMT programs and encourage further research within this population.

## Abbreviations

CATC: Canadian Addiction Treatment Centres
GENOA: Genetics of Opioid Research
MMT: Methadone maintenance treatment
OUD: Opioid use disorder
PO: Prescription opioids
